# The Longitudinal Association of Walking Efficiency With Brain Volumes in Community-Dwelling Older Adults

**DOI:** 10.1093/geroni/igaa057.2834

**Published:** 2020-12-16

**Authors:** Jennifer Schrack, Fangyu Liu, Amal Wanigatunga, Yang An, Christos Davatzikos, Eleanor Simonsick, Luigi Ferrucci, Susan Resnick

**Affiliations:** 1 Johns Hopkins Bloomberg School of Public Health, Baltimore, Maryland, United States; 2 National Institute on Aging, Bethesda, Maryland, United States; 3 University of Pennsylvania, Philadelphia, Pennsylvania, United States

## Abstract

Walking efficiency (WE) predicts mobility decline and is linked with higher fatigability. Fatigability is associated with cognitive decline and reduced brain volumes (BV), but the link between WE and BV is undefined. We examined associations between WE and BV in 860 participants of the BLSA (mean age 66.4(14.4) years, 54.5% women). WE was assessed during 2.5-minutes of usual-paced walking using indirect calorimetry and standardized per meter (ml/kg/m). BV measures were derived using MRI scans and an automated multi-atlas region-of-interest approach. In linear mixed models adjusted for demographics, education, BMI, intracranial volume, and cognitive status, lower baseline WE was associated with lower total, white, and gray matter, primarily in the frontal and temporal lobes (all p<0.05). Longitudinally, declining WE was associated with increasing ventricular and decreasing hippocampal volumes over follow-up (all p<0.01). Findings suggest rising age-related inefficiencies may reflect underlying brain atrophy and serve as a novel indicator for future interventions.

